# Job Demands and Resources During Digital Transformation in Public Administration: A Qualitative Study

**DOI:** 10.3390/bs16020187

**Published:** 2026-01-27

**Authors:** Victoria Sump, Tanja Wirth, Volker Harth, Stefanie Mache

**Affiliations:** Institute for Occupational and Maritime Medicine (ZfAM), University Medical Center Hamburg-Eppendorf (UKE), Seewartenstraße 10, 20459 Hamburg, Germany; v.sump.ext@uke.de (V.S.); wirth.ext@uke.de (T.W.); harth@uke.de (V.H.)

**Keywords:** digital transformation, public administration, job demands, job resources, workplace health management, psychological well-being, workplace well-being, health-promotion

## Abstract

Digital transformation poses significant challenges to employee well-being, particularly in public administration, where hierarchical structures, increasing digitalization pressures, and high mental health-related absenteeism underscore the need to understand individual and job demands and resources. This study explores these aspects from the perspectives of employees and supervisors in public administration. Between September 2023 and February 2024, semi-structured interviews were conducted with eight employees and eleven supervisors from public administration organizations in Northern Germany and analyzed using deductive–inductive qualitative content analysis based on the Job Demands-Resources model. Identified individual resources included technical affinity, error tolerance, and willingness to learn, while key job resources involved early and transparent communication, attentive leadership, technical support, and counseling services, with most job resources linked to leadership behavior and work organization. Reported job demands comprised insufficient participation, inadequate planning, and lengthy procedures, whereas personal demands included fears and concerns about upcoming changes and negative attitudes toward transformation. The variation in perceived demands and resources highlights the individuality of the employees’ experiences. The findings provide initial insights into factors influencing psychological well-being at work during digital transformation, emphasizing the importance of participatory communication, employee involvement, leadership awareness of stressors, and competence development. Future research should employ longitudinal and interventional designs to improve causal understanding and generalizability.

## 1. Introduction

Digital transformation represents a critical challenge for German public administration. Despite Germany being the largest economy in the European Union (EU) (Destatis, 2025), it ranks only 18th out of 27 EU countries regarding the availability of digital public services (European Commission, 2022). The discrepancy underscores the urgent need for comprehensive digitalization efforts. Digital transformation involves fundamental structural and cultural changes necessary to integrate new digital technologies and digitalized processes successfully (Nadkarni & Prügl, 2021). Such organizational changes disrupt existing workflows and modify job demands (Schlicher et al., 2022), yet they also offer employees opportunities for skill development, greater autonomy, and flexible working arrangements (Beermann et al., 2018).

Klenk et al. (2020) frame digital transformation within public administration as a change management process, emphasizing the requisite cultural adaptations. Change management encompasses the systematic planning, implementation, management, and evaluation of change initiatives aimed at fostering employee acceptance (Schlicher et al., 2020). Prior research indicates that organizational change can be perceived as stressful or threatening, often resulting in adverse health outcomes, such as emotional exhaustion or burnout (Bamberg et al., 2022; Day et al., 2017; Dubois et al., 2013). Consequently, digital transformation may similarly impact employee well-being and psychological well-being at work. In addition to change-related stress, the digitization of work itself can act as a stressor, commonly conceptualized as technostress (Dragano & Lunau, 2020). Technostress refers to individuals’ difficulties coping with information and communication technologies (ICTs), leading to stress, exhaustion, and burnout (Dragano & Lunau, 2020). Against this background, the present exploratory qualitative study does not aim to assess stress or technostress as outcomes. Instead, it focuses on identifying demands and resources associated with digital transformation in public administration in order to understand factors that may potentially contribute to work-related strain or support employees’ psychological well-being.

Approximately 10% of public administration employees have been identified as experiencing significant digital stress (Wrede et al., 2021). This subgroup typically exhibits lower support for digitalization, perceives insufficient employer support, reports stress from prolonged screen time and digital availability demands, shows reduced job satisfaction, lacks social support, experiences higher turnover intentions, and is more frequently overwhelmed. Demographically, digitally stressed employees tend to be older and possess lower educational attainment. Beyond digital stressors, the public administration sector faces elevated rates of sickness absence, notably due to mental health disorders. In 2022, it recorded the second-highest number of absence days related to mental health disorders in Germany, second only to the healthcare sector (DAK, 2024). These figures illustrate the vulnerability of the workforce, although the present study focuses on work-related demands and resources rather than clinical outcomes.

Thus, safeguarding employees’ psychological well-being in the workplace amid ongoing digital transformation and demographic challenges, such as retirement associated with an aging workforce (Reichard & Schröter, 2021), is necessary. To maximize the benefits of digital transformation while mitigating its risks, a nuanced understanding of demands and resources emerging in this context is essential (Schlicher et al., 2022). Identifying these factors enables the design of targeted, demand-oriented change support interventions, which are likely more effective than generalized approaches (Schlicher et al., 2022).

## 2. Background

From a theoretical perspective, working conditions in general can be categorized into job demands and job resources, as proposed by the Job Demand-Resources (JD-Rs) model (Bakker & Demerouti, 2007). Job demands (referred to as demands in the following) are defined as “physical, psychological, social and organizational aspects of the work, which usually require a physical and/or mental strain over a longer period of time, and are therefore associated with certain physiological and/or psychological costs” (Demerouti & Nachreiner, 2019, p. 121). On the other hand, there are job resources (referred to as resources in the following), which contribute to the achievement of work-related goals, the reduction in demands, their consequences, and personal development; therefore, they “can play an active role in the prevention of exhaustion” (Xanthopoulou et al., 2007, p. 136). The JD-Rs can be extended by personal demands and personal resources. Personal resources include self-efficacy, organization-based self-esteem, and optimism, which mediate the relationship between job resources and work engagement (Xanthopoulou et al., 2007). Personal demands, i.e., workaholism and perfectionism, can be understood as “the requirements that individuals set for their own performance and behavior that force them to invest effort in their work and are therefore associated with physical and psychological costs” (Barbier et al., 2013, p. 75). The dynamics of demands and resources can either be associated with motivation or with reduced health and an increased risk of burnout when high demands are not accompanied by resources (Demerouti & Nachreiner, 2019). Thus, the JD-R model (Bakker & Demerouti, 2007) provides an appropriate theoretical background for the dynamic working conditions in digital transformation and might be “particularly useful in understanding the impact of technology and digital transformation on work-related wellbeing” (Trenerry et al., 2021, p. 10).

### 2.1. Demands in Digital Transformation

To our knowledge, there are only a few studies focusing on demands, resources, and outcomes that arise within digital transformation in public administration. According to a systematic review by Wirth and Mache (2023), demands on employees in the context of digital transformation can be allocated to work organization (i.e., increased work load, lack of role clarity, job uncertainty), work content (i.e., insufficient communication, concern of quality losses), and leadership (i.e., insufficient planning, lack of engagement, lack of support of employees). Further specific digital demands can be classified as availability via ICT, dependence on ICT, work intensification, increased expectations regarding learning, and insecure environments that are linked to digital transformation (Scholze & Hecker, 2023). Time pressure, work intensification, constant need to adapt to changing demands, and an increase in demanding tasks are further demands identified in the context of digitalization (Bretschneider et al., 2020).

### 2.2. Resources in Digital Transformation

On the other hand, specific resources can arise in digital transformation. Transparency of digital transformation processes, active participation of employees, technical support, support provided by supervisors, and individual competence development have been identified as relevant resources in the context of digital transformation (Wirth & Mache, 2023). The development of digital competences is relevant to cope with digital demands and prevent digital overload (Wrede et al., 2021). Digital resources may further include autonomy through ICT, collaboration facilitated through ICT, and efficiency gains through ICT (Scholze & Hecker, 2023). In addition, a positive attitude towards digitalization and individual adaptability can represent a personal resource in digital transformation (Trenerry et al., 2021). Workplace resilience represents another potential resource in digital transformation contexts, as also highlighted by recent qualitative research on psychological resilience and digital transformation (Sayed Alitabar & Parsakia, 2025; Trenerry et al., 2021). Resilience, understood as the ability to cope with adversity (Harms et al., 2017), is influenced, i.e., by personality traits and cultural values (Wei & Taormina, 2014) and is linked to the ability to manage job demands (Kinman & Grant, 2011). Fostering these factors might support employees by promoting psychological well-being at work and job satisfaction, thereby helping them to cope with demands arising from digital transformation. Leadership behavior can be understood as a central contextual factor influencing how demands and resources are shaped during digital transformation processes. In situations characterized by increased demands and uncertainty, supervisors’ self-reports of their own behavior provide insights into how leadership intentions and self-perceptions guide communication, participation, and support during change. Prior research highlights self-reflection and self-awareness as key mechanisms of self-regulation that inform supervisors’ leadership behavior in demanding work environments (Bandura, 1991; Hannah et al., 2011). From this perspective, supervisors’ self-referential accounts offer a theoretically grounded basis for examining leadership-related demands and resources in digital transformation contexts.

### 2.3. Aims

To date, there is a lack of empirical evidence regarding the specific demands and needs arising from digital transformation within public administration (Schlicher et al., 2022). Public administration is characterized by specific organizational and cultural features such as silo structures, a strong resort principle reflected in clearly separated departmental responsibilities, and hierarchization (Klenk et al., 2020; Christensen & Lægreid, 2007). In addition, the multilevel system of Germany’s state and municipal public administration supposedly impedes decision and implementation processes in digital transformation (Mergel, 2021). Therefore, transferability of results in the context of digital transformation in the private sector, as described previously, might be limited in terms of the context of public administration.

This study aims to address this gap by expanding the understanding of specific demands (e.g., job insecurity, inadequate communication) and resources (e.g., competencies, organizational support) that emerge during digitalization in public administration. A thorough understanding of these demands and resources is essential to foster employee well-being and mitigate negative health- and work-related outcomes.

The central research question guiding this study is the following:

“What individual and job demands and resources did employees and supervisors in public administration experience during prior digital transformation initiatives?”

To address this question, we assessed the experiences of employees and supervisors (i.e., employees holding formal managerial responsibility in the hierarchical structure of public administration) regarding demands and resources encountered during past digital transformation within public administration. Our goal is to contribute to the identification of change demands and change resources specific to this context, thereby informing the development of targeted, demand-oriented change support interventions.

## 3. Materials and Methods

### 3.1. Study Design

This qualitative, exploratory study employed semi-structured, guideline-based interviews following Witzel’s (1985) Problem-Centered Interview (PCI) methodology. A qualitative approach was chosen as it is particularly suited for generating in-depth insight in an emerging field of research (Mayring, 2015). The study adhered to the Consolidated Criteria for Reporting Qualitative Research (COREQ) (Tong et al., 2007), as well as established standards for qualitative research reporting (Mayring & Fenzl, 2022). The completed COREQ checklist is available in Supplement Material S1. Ethical approval was obtained on 5 June 2020 from the Local Psychological Ethics Committee (LPEK) at the Centre for Psychosocial Medicine at Hamburg University Hospital (reference LPEK-0167), and the study was conducted in accordance with the Declaration of Helsinki.

### 3.2. Recruitment and Participation

Eligibility criteria for participation in the interviews included the following: (1) age over 18 years, (2) current employment within public administration, (3) a minimum employment duration of six months in public administration, and (4) at least part-time employment (≥19 h per week). Potential participants were initially identified through an internal mailing list and subsequently contacted randomly via E-Mail (*n* = 24), which included an informational flyer detailing the study’s content and objectives. Additional participants were recruited using the snowball sampling technique (*n* = 3). Of those 27 individuals contacted by E-Mail, 70% consented to participate, resulting in a final sample of 19 participants. Efforts were made to ensure participants represented a diverse range of authorities within public administration to enhance the transferability of findings. Participants were drawn from administrative or central departments across 11 different authorities or institutions; to preserve anonymity, specific entities are not disclosed. Experience with any prior digital transformation sufficed for inclusion with participant experiences ranging from softphone hardware implementation to software migration and large-scale software implementation.

Participation was voluntary, and no financial compensation was provided. Prior to the interview, all participants received written information outlining the purpose of the study, the measures taken to ensure confidentiality, and how data would be handled. Written informed consent was obtained from all participants before data collection began.

### 3.3. Guidelines

The interview guidelines were developed in collaboration with the second author (SM). They began with an introduction informing participants briefly about the study’s purpose, aims, and the interviewer’s role. Prior to the interview, sociodemographic data were collected using a short paper-and-pencil questionnaire to ensure anonymity. The core of the guidelines consisted of three thematic blocks with open-ended questions addressing the following: (1) experiences of opportunities (resources) and challenges (demands) in previous digital transformation, (2) anticipated opportunities (resources) and challenges (demands) in future digital transformation, and (3) needs related to future digital transformation. Only the first thematic block is analyzed in this study (see Appendix A Table A1); the remaining parts will be addressed in a separate publication and are therefore not discussed here. Participants were asked to reflect on their individual experiences rather than on a specific digital transformation. The questions about opportunities and challenges were based on the JD-R model (Bakker & Demerouti, 2007) and previous findings (e.g., Wirth & Mache, 2023). To facilitate a more accessible conversation, the terms “opportunities” and “challenges” were used instead of the technical terms “resources” and “demands”, as the assessment of these concepts was implicit (see Table 1).

A pre-test was conducted with *n* = 2 interviewees by the first author (VS) to test comprehensibility and usefulness of the guidelines; adjustments were made based on the feedback. These interviews were not included in the present study.

### 3.4. Categories

For the deductive analysis, an initial coding system was developed based on the theoretical framework and the content of the interview guide (see Table 1). Drawing on organizational behavior frameworks (Wagner & Hollenbeck, 2010), demands and resources were categorized into four levels: (1) personal factors, (2) group factors, (3) leadership factors, and (4) organizational factors (Schaufeli, 2017; Wirth & Mache, 2023). Additionally, the Technology Acceptance Model (TAM) has been used in prior research to conceptualize why technology-related aspects, such as perceived usefulness and ease of use, are relevant for the adoption of digital technologies (Davis, 1989). In the present study, TAM was not applied as an analytical model, but served as a theoretical reference to justify the relevance of technology-related demands and resources. Accordingly, this study places particular emphasis on demands and resources related to technology use. These technology-related demands and resources were further assigned to a fifth category: (5) work organization and content, as proposed by the Joint German Occupational Safety and Health Strategy (GDA) (GDA—Arbeitsprogramm Psyche, 2022; Beck et al., 2023).

### 3.5. Data Collection

Data were collected from employees and supervisors working in public administration using two slightly adapted interview guidelines tailored to the participants (see Table 1). In this study, supervisors were defined as employees holding formal managerial positions with supervisory and personnel responsibility within public administrative organizations. These supervisors were responsible for teams or organizational units based on their formal role within the organizational hierarchy rather than informal influence. All supervisory positions represented administrative roles filled through standard recruitment procedures and were neither politically appointed nor elected. To ensure the highest quality and depth of information, interviews were primarily conducted one-on-one in a face-to-face setting. However, due to sudden illnesses (*n* = 4) and for geographic reasons (*n* = 1), a total of five interviews were conducted online via Skype. Face-to-face interviews took place at either the participants’ work site (*n* = 13) or at the interviewers’ office (*n* = 1). All interviews were conducted on a single occasion between September 2023 and February 2024 by VS in northern Germany. The interviewer (VS) is a female psychologist and employed researcher in this field of occupational medicine, working in a research group focusing on mental health at the workplace, and was familiarized with established quality standards and procedures for qualitative interviews (Helfferich, 2011). This professional background may have shaped the focus of the interviews and the iterative analytic process, including coding and category development, which was continuously reflected upon through reflexive team discussions. All interviews were audio-recorded and lasted between 40 and 64 min, with a total recording time of 960 min (16 h). No interviews were repeated. The recurring mention of demands and resources across indicated that theoretical saturation was achieved (Strübing, 2018).

### 3.6. Data Analysis

The interviews were transcribed using a two-step process. First, automatic speech recognition software f4x (Version 2023, Dr. Dresing & Pehl GmbH, Marburg, Germany) was used to generate initial transcripts. These were subsequently manually corrected, anonymized, and finalized by the first author (VS). Transcripts were not returned to participants for member checking.

Sociodemographic data were analyzed using Microsoft Excel 2019 (Redmond, DC, USA). Qualitative data analysis was conducted with MAXQDA 2022 (VERBI Software, 2022) following qualitative content analysis guidelines that combined deductive and inductive approaches (Mayring & Fenzl, 2022). No interviews were excluded from the analysis.

Initially, all transcripts were read and coded according to the predefined code system. After coding approximately half of the transcripts, coded segments were reviewed to ensure linguistic accuracy and discrimination. This deductive coding was complemented by an inductive approach, which involved extracting additional expressions related to demands and resources across the five predefined levels of digital transformation.

An iterative process of category, subcategory, and code refinement was employed until no new categories emerged, indicating data saturation. Following Mayring’s (2015) qualitative content analysis method, all coded segments were generalized and condensed to structure the content further, without paraphrasing due to the volume of data.

The categorization logic and final category names were discussed and agreed upon with the second author (SM). To illustrate the findings presented in the results section, selected quotes were translated from German to English. A selection of further relevant quotes is available in Supplement Material Table S2, without claim to completeness.

## 4. Results

### 4.1. Sample Description

A total of 19 participants took part in this study, comprising 8 employees and 11 supervisors. The majority identified as female (63.1%), with one participant identifying as non-binary (5.3%). Further sociodemographic details are provided in Table 2. Most participants were civil servants (52.6%), with a notably higher proportion among supervisors (81.2%) compared to employees (12.5%).

### 4.2. Job and Personal Demands and Resources Perceived in Previous Digital Transformation

In accordance with the JD-R model (Bakker & Demerouti, 2007), different resources and demands were experienced and reported in previous digital transformation processes by employees and supervisors within the public administration. As described before, demands and resources were assigned to personal, group, leadership, and organizational factors, as well as work organization and content (Nielsen et al., 2018). No demands related to group factors were mentioned.

### 4.3. Personal Resources and Job Resources Perceived in Previous Digital Transformation

#### 4.3.1. Personal Resources in Previous Digital Transformation

Openness towards change and digital transformation: The personal attitude towards change (openness) and one’s own acceptance of change were perceived as helpful by a few employees and supervisors to be able to engage in change processes.

*“So, I think a lot of it always depends on whether I get engaged. So, that also has something to do with change. First of all, ‘okay, everything’s going well, why should we have something new now?’. Then I always think, ‘This has been developed, someone has thought about it, I’ll accept it now, I’ll go for it’. So, the personal attitude of whether I accept it, is, I think, very, very important.” (Participant 14, female employee, age 51–60)*

Technical affinity: Some employees and supervisors expressed that growing up with technology has helped them adapt and acquire knowledge quickly during previous digital transformations. Consequently, they stated that they were less afraid of technology compared to older generations. Their technical affinity enabled them to adapt to change more easily and was therefore perceived as an individual resource.

*“I think I grew up with technology.(…). I didn’t learn to type on a typewriter, and I think that’s the reason you have less fear and respect for technology.” (Participant 19, female employee, age 21–30)*

Error tolerance and willingness to learn: The acceptance and tolerance of one’s own mistakes in dealing with new programs, the willingness to learn new things, and self-efficacy expectation regarding learning were perceived as helpful in previous digital transformation by several employees.

*“When I must deal with it, at the beginning, it’s a bit cumbersome, but I can trust it, I’m still young enough (…) and I can learn it. I also try things out and I’m not going to shut down the whole [city] if I click on the wrong button. So that’s really the feeling some people have when I click something wrong, I don’t have that, but the system somehow forgives me most of the time.” (Participant 24, non-binary employee, age 51–60)*

Further personal resources: Further personal resources that were mentioned by a few employees were the ability to recognize the personal added value in digital transformation and the ability to identify decision-making leeway in digital transformation:

*“To be honest, I’m really good to find something stupid at first and then looking to see what’s good about it, and in case of doubt, what’s good about the bad. And when it comes to digital changes, I’m at least good at recognizing where the benefit is for me.” (Participant 24, non-binary employee, age 51–60)*

#### 4.3.2. Resources Related to Group Factors in Previous Digital Transformation

Positive team atmosphere: A few employees perceived a positive atmosphere in the team as relevant for increasing motivation and involvement in the process and for planning any additional work or preparatory measures together.

*“So, the whole process is exciting. I also found the project work itself really exciting, you get a bit of a glimpse of it. And I already knew the project manager from a previous training course, and I thought she was really, really nice and I thought, ‘Yes, that might be a really cool way to work together’. I think it might have been a bit different if the atmosphere [in the team] had been different.” (Participant 19, female employee, age 21–30)*

Social support: Several employees and supervisors perceived support from colleagues (e.g., regarding technical questions) and within the team as helpful in previous change processes.

*“There are days when I don’t know how to start. And then I see my colleague and her hair stands on end. Then we make nonsense together and then we feel much better again. So, for me personally, the team and my colleagues are a huge protective factor in being able to work well.” (Participant 14, female employee, age 51–60)*

#### 4.3.3. Resources Related to Leadership Behavior in Previous Digital Transformation

In this section, the perspectives of employees on helpful leadership will be compared to the perspectives of supervisors on their own leadership and its effect on employees.

Most employees perceived leadership as supportive in digital transformation under the following circumstances:

Supervisors’ positive attitude towards change: Leadership behavior was perceived as supportive when the supervisor brought change to the team in a positive way, as they had a positive attitude towards change themselves. This was described as helpful in convincing the employees of the need for change.

*“She was also very positive and convinced the team and got us all on board. But of course, you can only do that if you think it’s good yourself.” (Participant 14, female employee, age 51–60)*

Understanding of different IT skills: Further, the supervisors’ understanding of different levels of IT skills of the employees (e.g., due to generational differences) was perceived as helpful, as well as support from the supervisor regarding IT-related questions. This was perceived as benevolent and supportive.

*“And I perceive her as very positive, that she (...) understands this generational thing, these differences. What I said at the beginning of the conversation about how we weren’t born, so to speak, if we absorbed that with our mother’s milk and I didn’t, the digital world, so she is very, very understanding and always open. So, if I were to say, ‘now I need this and that training’, then I would definitely get her support. (…) But she’s one of those people who I think I ask for the fourth time, ‘Sorry, can you explain that to me again?’, and then she does it. So, she’s very benevolent and very supportive.” (Participant 27, female employee, age 51–60)*

Awareness and understanding of intensive preparation: supervisors actively supporting employees with preparatory measures for digital transformation were perceived positively. At the same time, supervisors’ understanding and awareness of time-consuming preparatory measures and any stress that may arise were mentioned positively.

*“For one thing, she is aware of how time-consuming it is. She also knows herself what’s lying dormant. (...) And so she also knows that it’s time-consuming and that we simply need this time, which we then take. (…) of course, she knows that this may be more stressful for them.” (Participant 16, male employee, age 41–50)*

Responsive and empathic contact person: Some supervisors expressed that being responsive to employees’ worries, concerns, and questions was experienced as supportive for employees. In this context, it was considered important to be patient as a supervisor and to have understanding and empathy for the various IT skills of employees.

*“Lots of patience and water. So really, sometimes I had fringes around my mouth. And above all, when you think, ‘Oh please, not that topic again’, but even the eighth time you have to listen to it in the corridor if necessary. So, patience in connection with a willingness to talk and definitely also structure.” (Participant 12, female supervisor, age 51–60)*

Open communication: An open exchange with employees and a general willingness to talk were also seen as important.

*“I mean, I tend to talk very openly with my colleagues. So, on the hiking side, of course you can’t always just say, ‘this is bad’, and ‘this is crap’, and ‘this is no good’, because that reinforces the whole thing. So, we had a very open exchange here about what wasn’t working well and then the colleagues, who were involved in these working groups reflected this back to the project.” (Participant 15, male supervisor age 61–70)*

Responsibility for employees’ capacities: As a supervisor, it was also seen as important to take care of employees’ capacities and to compensate them for overtime.

*“But we had underestimated the effort for [Name of Process], for example. (…) But it turned out relatively quickly that that wasn’t enough and then we had to take countermeasures (...). And then also to make it clear that the colleagues would be freed up from their normal day-to-day business, so to take appropriate care of them.” (Participant 12, female supervisor, age 51–60)*

#### 4.3.4. Resources Related to Organizational Factors in Previous Digital Transformation

General Counseling service: Few employees expressed that offering counseling services has helped employees reduce worries and fears regarding change processes and fostered acceptance of change. Employees who rejected change in the first place were supported through counseling.

*“For one person, this change was quite terrible for them. And we were able to work on this quite well so that she could learn “Yes, this is a change now, but it’s okay not to fully understand everything straight away and it’s okay to make mistakes and it’s okay to perhaps have to consult with her boss more”. (Participant 24, non-binary employee, age 51–60)*

Central responsible contact person: Few supervisors perceived having a responsible person for the implementation of digital transformation as supportive in previous digital transformations.

*“It helped me a lot that there was a person responsible for the introduction of this application, and for the further development of this application. And it helped me a lot that this was a very committed person who managed to keep me very interested in this topic the whole time.” (Participant 23, male supervisor, age 51–60)*

#### 4.3.5. Resources Related to Work Organization and Content in Previous Digital Transformation

Efficiency of work processes: Some employees and supervisors expressed that through previous digitizing processes, efficiency (e.g., through automation) and transparency of processes increased, while resources could be saved (e.g., time savings, paper). This was perceived as a facilitation of work.

*“You no longer need all that paper, you no longer must wait, and you know who to speak to if it doesn’t go anywhere, you can see who the last person was to do it. You can read the notes that people write. There used to be handwritten notes and then you’d sit there and think, ‘What did the office manager write there? I can’t read it.’ (....) It’s more transparent, it’s more comprehensible.” (Participant 13, female employee, age 51–60)*

Supervisors added that through previous digital transformation, employees had more time resources for the core competencies, which was described as a relief for employees. In addition, the increased quality of work and reduction in errors were mentioned by supervisors as a relieving factor.

*“For the employees here, that say, ‘I am happy that I only have that, I only get the results and don’t have to worry about the quality’, because the quality is now produced by a robot and time resources are available for employees.” (Participant 7, male supervisor, age 41–50)*

Flexibility of work: Several employees and supervisors perceived the improvement in the flexibility of work (e.g., working remotely is made easier) as an opportunity from previous digital transformations.

Supervisors specifically added that through increased flexibility of work motivation of employees could be fostered and sick leave could be reduced.

*“Furthermore, it is important for us to be able to increase work life and family balance for our employees since digitalization and automatization enables our employees to work from home more frequently. For example, the topic [name of application], then you don’t have to be on site. Thereby we can foster motivation and were able to reduce sick leave significantly.” (Participant 2, female supervisor, age 41–50)*

User-friendly design of work content: Few employees perceived the user-friendly interface as positive. Tools with few predetermined breaking points and media interruptions (i.e., printing digitalized materials) were also perceived as positive and increased satisfaction with the tools.

*“It is a nice interface, so user-friendly.” (Participant 14, female employee, age 51–60)*

Participation: Some employees perceived it as supportive that they were able to make contributions, i.e., comments and ideas, e.g., via queries in the development of tools and processes in previous digital transformation, and that attempts were made to take these aspects into account. Even after the implementation of digitalized processes, the exchange, e.g., via user forums and opportunities for participation, was perceived as supportive.

Many supervisors mentioned that they experienced the broad participation of future users across hierarchies as supportive to integrate users’ needs in product and process development. Supervisors perceived participation as helpful to foster the openness of employees towards change and willingness to learn.

*“My impression was that we came together at the working level with the people involved, with many different players who had to work with this application. And the group defined the requirements for these new specialist applications. Then, in the course of the introduction of this ongoing project, there was a regular exchange between this group of users, both employees and supervisors who were involved, and then also a regular exchange with the company itself, which programmed this specialist application, in order to then define these things even more clearly in the form of workshops, ‘what exactly is needed’.” (Participant 23, male supervisor, age 51–60)*

Technical support: Personal support at the workplace (e.g., from HR departments), ongoing user support such as digital lookup or reading options (intranet, digital manuals), events, and telephone contacts in the IT departments were perceived to provide support with technical issues by several employees and supervisors.

*“So, there is permanent support that you can fall back on by looking up: what do I need? And then I look it up briefly, as there are also offers from time to time (..) and certain IT formats are also offered there, but I think these small, these little bites are much better.” (Participant 27, female employee, age 51–60)*

In addition, supervisors mentioned floorwalkers or multiplicators as supportive in previous digital transformations.

*“There were so called floorwalkers, that went to the people and sat down with them, that you had direct multiplicators for the daily work. That is of course a great thing.” (Participant 01, female supervisor, age 41–50)*

Early and transparent communication: Many employees and supervisors perceived the early and transparent communication in previous digital transformations as supportive. The early communication of upcoming changes (e.g., through newsletters) has resulted in employees being open to them and changes being well-received. By communicating the added value of changes, personal benefits for employees could be highlighted. Continuous communication during the process was perceived as supportive. The transparent communication of challenges was also perceived as helpful.

*“Total transparency. As an authority, as [name of department], we always knew where the problems lay with the manufacturer, where the problems lay in the process, where the problems lay in the financing. So, it was really, at least that’s my feeling, it was completely open and transparent, everything was explained, and you knew where you stood. (…) And that are things that you expect a bit, that consequently foster acceptance.” (Participant 15, male supervisor, age 61–70)*

Individual competence development: Several employees and supervisors perceived training and testing of the user interface prior to software introduction or renewal as supportive. This was perceived as helpful to alleviate concerns and fears. Continuous (low threshold) training or support offers (user forums, IT knowledge formats) that also existed after the introduction were perceived as helpful.

*“We had the opportunity to have such a dummy environment in the training course. So that you can see what it actually looks like. Because one thing is to talk about it (…) or I see how it actually works. And I think that also helped to break that down. It [concerns and fears] certainly hasn’t gone away completely. I don’t think it has to this day, because it’s different and it’s a change. (...) But I think we were definitely able to reduce it.” (Participant 19, female employee, age 21–30)*

#### 4.3.6. Analytical Summary

Resources experienced during previous digital transformation processes were reported across personal, group-related, leadership-related, organizational, and work organization and content-related levels. Leadership-related resources were reported as particularly salient, while group-related, organizational, and work-related resources were described alongside individual resources.

**Figure 1:**
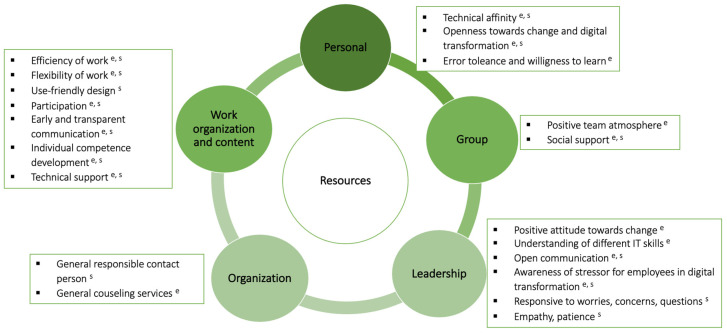
Personal and job resources that were mentioned by employees and supervisors in previous digital transformations in public administration. Notes: ^e^ = indicates that employees mentioned the experience of the respective resource; ^s^ = indicates that supervisors mentioned the experience of the respective resource.

Figure 1 provides an overview of the identified resources, which are described in detail in the preceding sections. The green circles indicate the categories to which the respective resources were assigned (see Section 3.4 for details).

### 4.4. Personal and Job Demands Perceived in Previous Digital Transformation

#### 4.4.1. Personal Demands in Previous Digital Transformation

Concerns and fears: On an emotional level, some employees described worries and fears as a challenge in previous digital transformations. Worries and fears were related to upcoming changes in general, lack of control over programs, making mistakes, and feeling helpless in the face of change. Rejection of change was described consequently.

*“And what I’ve already said is, that there’s also a lot of fear of change or of anything new, because it’s not predictable at first.” (Participant 27, female employee, age 51–60)*

Some supervisors experienced employees’ worries and fears as challenging. They added the worries of additional work and worries of employees being replaced by technology.

*“A lot of people are afraid of what is coming because they don’t know how to deal with IT. So, it is not uncommon for us to have areas where processes have been the same for 40 years (….). Employees are afraid to be outdated, to not be able to do their jobs, that they don’t understand the technology. They are afraid that they are going to lose their job, which hasn’t happened, or that they are simply overworked and that it will be more work for them; that is a very big issue for them.” (Participant 21, female supervisor, age 21–30)*

Learning new IT skills: Few employees, who ascribed themselves to the older generation, expressed having difficulties acquiring new digital know-how quickly and sustainably in a previous digital transformation.

*“() … and I don’t want to say the older generation, but I think I have to classify myself a bit like that. And I have to say quite clearly that my two colleagues are in their early 30s, and I’ve really noticed the difference. They grew up with social media, smartphones, and so on (…). And then if I haven’t done it for three or four weeks, I must think, ‘What were the steps again’? And it was very painful to realize this difference, which still existed. (…) I still need support and that really annoys me. But that’s my challenge and the challenge of everyone in my age group.” (Participant 27, female employee, age 51–60)*

Lack of IT skills: A few supervisors reported that their employees seemed overworked as a result of inadequate IT competencies. In addition, supervising these employees demanded greater effort from the supervisors.

*“I was shocked, but a few years ago, a [name of support service] had to teach an employee how to use a mouse and a keypad. I think that was a single case (..), but still these cases happen. And there are employees that partially don’t know how to use [name of program] and have to learn it here from the start. So, these are topics that are difficult regardless of the digitalization projects and were the basic requirement are really lacking.” (Participant 21, female manager, age 21–30)*

Negative attitude towards change: A few supervisors experienced employees’ negative attitudes towards change as a challenge. Consequently, supervisors perceived their employees as stressed and had to make significant efforts to motivate and win their commitment.

*“And I can actually think of one colleague who always seemed to see the glass half empty and, to be honest, always almost empty, not just half empty, and that’s generally difficult. Those are really the challenges where you have to go back and talk again or take the time to talk. That was difficult until the end.” (Participant 12, female supervisor, age 51–60)*

#### 4.4.2. Demands Related to Leadership Behavior in Previous Digital Transformation

Negative attitude towards change: A few employees described supervisors’ negative attitudes towards change or resistance to change as a challenge in previous digital transformations.

*“(…) there are also line supervisors who, for whatever reason, simply don’t see that yet or whatever, and might also block them a little under certain circumstances.” (Participant 16, male employee, age 41–50)*

Inattentiveness to support needs of employees: A few supervisors described the inattentiveness of supervisors towards employees’ needs as a challenge, as they recognized their employees’ needs too late or did not consider themselves responsible for addressing them.

*“Because (...) I am of the opinion that managers have not looked closely enough and have not recognized early enough when support is needed, when a support offer is needed or when we need to have a conversation about what is the reason for not being able to deal with the digital way of working? Where is a need for training?” (Participant 23, male superviso, age 51–60)*

Moodiness: Few supervisors expressed that they were afraid to ask questions in previous digital transformations due to their own superiors’^1^ moodiness (choleric).

*“It sounds so stupid, but if you’re afraid to go into an office because you think ’Oh, she’s in a bad mood again, I’m not going to go in there’. Or you have questions, and you don’t know how to deal with them (...) then of course that’s super counterproductive.” (Participant 17, female supervisor, age 21–30)*

#### 4.4.3. Demands Related to Organizational Factors in Previous Digital Transformation

Decision-making bodies impede processes: Some supervisors expressed that decision-making bodies (i.e., personnel council) impeded digital transformation projects, partially against the employees’ wishes.

*“In some cases, this fails due to data protection or staff councils, i.e., co-determination bodies, which do not understand or sometimes block it out of false ambition, sometimes against the wishes of the employees.” (Participant 25, male supervisor, age 41–50)*

Insufficient planning: Some employees and supervisors described the following challenges that can be ascribed to the planning and implementation of change processes:

- Additional work due to postponements of projects, which was experienced as annoying.
- Disappointment due to the postponement or cancellation of projects due to a lack of planning or coordination.
- Insufficient planning.
- Announced projects are postponed to unknown.
- Lack of assignment of roles and responsibilities.
- Lack of dissemination of processes by supervisors, and in general, led to resentment among employees.

*“As the project was postponed from time to time, we had to start again two or three times. I think that was also the most annoying part of the preparation. Because we started it once, then we were told to postpone it again, then, the demands were different again.” (Participant 19, female employee, age 21–30)*

Long procedure: Few supervisors experienced the length and duration of previous digital transformations as challenging, as knowledge and many decision makers are involved.

*“I know the lead times of projects at [Authority], especially when it comes to the introduction of software products, I would say quite bluntly, nothing takes less than five years, and it can easily become ten.” (Participant 12, female supervisor, age 51–60)*

Lack of resources (personal and financial): Few supervisors complained that due to a lack of personnel resources, digital transformation could not be realized in previous transformations; this has led to resentment. A few supervisors and employees expressed that in previous digital transformations, there was a lack of financial resources to provide high-quality programs or tools. Extra work was needed to compensate for missing features or quality. Aside from that, they mentioned a lack of financial resources to cover support for the entire project duration.

*“(…) The tools that provide the best solution and are more expensive are usually not chosen, but always the cheaper ones and they are just crutches. So, in the end, we spend more time with it, because the personnel costs, the hidden costs, are not included there and you have a lot of people who have to deals with that, because it is so bad, that in the end it turns out more expensive.” (Participant 02, female supervisor, age 51–60)*

#### 4.4.4. Demands Related to Work Organization and Content in Previous Digital Transformation

Insufficient or lack of participation: Many employees and supervisors described the insufficient or lack of participation of employees as a challenge in previous digital transformations. From the employees’ point of view, this has led to a lack of functions in implemented programs. Employees felt left alone, overwhelmed, and frustrated as a reaction to the lack of involvement. This has resulted in a negative attitude toward the newly introduced software, leading to a disorganized implementation process. The involvement of older employees in change processes was described as an additional challenge. They showed more skepticism, which had to be countered accordingly. It was also perceived negatively when feedback on missing functionalities was ignored.

*“And the employees weren’t really involved in the whole process. When it was suddenly said: Yes, here it is, have fun with it now. (…..) But as I said, many of them simply felt left alone and were a bit outraged and frustrated. It was a mixture of feelings, but they didn’t like it. So, I don’t think they really gave the software a chance. For the most part, it was very user-friendly, which is also important. I actually find it very intuitive, but because they were simply left alone so much from the start, they didn’t really give it a chance.” (Participant 4, female employee, age 31–40)*

In addition, few supervisors mentioned the pseudo-participation of employees as a challenge in previous processes. In practice, this meant that although employees were formally involved and provided feedback, their contributions were not implemented, ultimately leading to software errors. This resulted in a loss of acceptance.

*“(...) On the other hand, the users on the part of the authorities, i.e., the case workers, are often not informed at all or they are tried to be involved, but in a pseudo-participation, where you don’t hear the hints because you say, ‘yes, but that’s too expensive’, ’It’s great that you want that, but we can’t do that. We have a STANDARD. It has to work’. As an example, [Name of Document], great fiasco. (…). In some cases, the processes for the inspectors have increased from 9 min to 13 min although relevant key users were involved and even though they had given enough indications that this would not work. Somehow it was not taken into consideration. I don’t know why, if it was money or why the project management just didn’t take it seriously, ‘these are problematic people’, there are always such things. But in retrospect, of course this leads to a huge loss of acceptance and frustration among employees.” (Participant 25, male supervisor, age 41–50)*

Digital transformation impedes work processes: Many employees and supervisors described that in previous digital transformations, malfunctioning technology, lack of access, or non-functioning programs and extension of process steps have led to extra work, frustration, stress, and strain. Processes that did not meet a user’s needs, were not fully developed, or could not be mapped digitally, were also described as a challenge. This led to extra work, and workarounds had to be found (i.e., manual calculation).

*“And certainly, the fact that this [data] was not recorded was just overlooked. So, it wasn’t thought through to the end. And, the problem is, well, I think that’s also legitimate, when you introduce innovations, then you also see first, you start sometimes where there’s a problem. So that’s also legitimate. It’s just that then it becomes a huge burden, because areas of work that are only marginally involved also experience an additional burden in their daily work, simply because here the business is so HUGE. In other words, you can’t just say, well, now you might have to enter certain [data] manually, because unfortunately things have gone wrong. We’re talking about, let’s say, male, female, non-binary has not been transferred. But then you must transfer it thirty thousand times. And then you have a completely different pile, (...) it always multiplies so incredibly.” (Participant 01, female supervisor, age 41–50)*

Insufficient or lack of communication: Few employees and supervisors mentioned that previous changes have not been communicated or reached employees, resulting in additional work.

*“From the IT department, I got the information that probably 80 to 90 percent of data they receive, is still the old form. It is not that we kept it a secret, but you can’t get it out of people’s heads. They still download the old version and save it on their desktop, no matter what. (….) Technology is ready, but I have not redirected people’s attention. (Participant 1, female supervisor, age 41–50)”*

In addition, some supervisors described a lack of communication or a lack of networks as a great challenge in previous digital transformations. This resulted in the authorities getting in each other’s ways and that work was conducted twice over.

*“(…) And any time I ask the question, ’You are aware that, this and that person, are working on it’, and then they look at me and respond, ‘No’, then I say, ‘Guys, you partly are one house/institution’. (…) I said,’ It feels like you are working in offices next to each other partly’ and said,’ You are not talking to each other’. That is a HUGE problem, we have from the city; not talking to each other.” (Participant 07, male supervisor, age 41–50)*

Integration of different demands: It was perceived as challenging by some supervisors that various requirements of different authorities had to be integrated into one process or application. Therefore, processes or applications did not meet all requirements.

*“(…) because the tool did not supply everything we need to work here. And that is of course, if there are tools that are for the whole city, that is of course basically right, because we all need the same things. Nevertheless, there are also special features in the detail.” (Participant 01, female supervisor, age 41–50)*

Legal regulation and data protection: A few supervisors perceived compliance with data protection regulations as challenging. In addition, employees’ legal concerns as to whether processes can be implemented as planned or not were mentioned.

*“Exactly, and there are often a lot of legal regulations, which is why a software idea may be wonderful, but cannot be implemented in practice. And employees naturally have a much better idea of what needs to be considered and is realistic. I often perceive employees worry that it simply can’t be implemented for legal reasons and then they are faced with this software.” (Participant 21, female manager, age 21–30)*

#### 4.4.5. Analytical Summary

Job demands during previous digital transformation processes were reported across personal, leadership-related, organizational, and work organization and content-related levels. Reported demands included emotional challenges, skill-related difficulties, organizational constraints, and disruptions of work processes.

**Figure 2:**
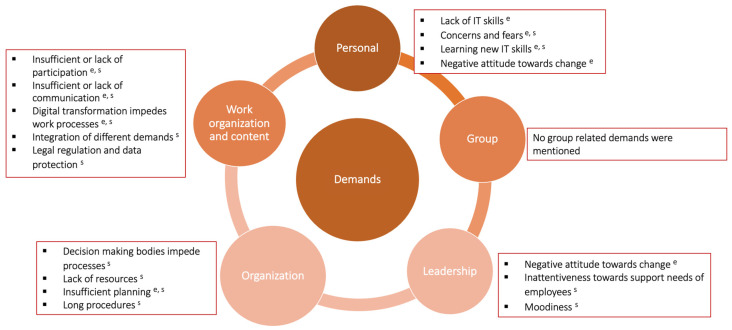
Personal and job demands that were mentioned by employees and supervisors in previous digital transformations in the public administration. Notes: ^e^ = indicates that employees mentioned the experience of the respective resource; ^s^ = indicates that supervisors mentioned the experience of the respective resource.

Figure 2 provides an overview of the identified demands, which are described in detail in the preceding sections. The red circles indicate the categories to which the respective demands were assigned (see Section 3.4 for details).

## 5. Discussion

Future research directions may also be highlighted. In the context of digital transformation in public administration, distinct challenges and opportunities arise that significantly affect employees’ psychological well-being at work and must be carefully considered when designing change management processes. The aim of this study was to identify both personal and job demands and resources experienced by employees and supervisors during previous digital transformation efforts within the public sector.

To our knowledge, this is the first empirical study to provide in-depth insights into these demands and resources from multiple perspectives within public administration. Our findings reveal a wide variety of demands and resources, highlighting the subjective and individual nature of employees’ experiences across different organizational levels. This underlines the importance of offering a broad spectrum of tailored support services to effectively promote and maintain psychological well-being at work during ongoing digital transformation.

Six key factors emerged as both potential demands and resources, depending on their presence or absence (see Table 3): (1) employees’ attitudes towards digital transformation or impending change, (2) supervisors’ attitudes towards digital transformation, (3) supervisors’ awareness (or lack thereof) of employees’ needs and stressors during digital transformation, (4) participatory communication, (5) employee participation, and (6) the impact of digital transformation on work efficiency. We propose that these factors play a crucial role in shaping health-promoting working conditions throughout digital transformation processes in public administration.

These findings align with the existing literature that emphasizes the influence of employees’ attitude towards digital technologies, their digital competences, participation in the implementation process (Zhang et al., 2023), and the availability of technical support on whether working with new technologies or software is perceived as stressful (Dragano et al., 2021; Wirth & Mache, 2023). Moreover, our findings correspond with an empirical online study on change management approaches in digital transformation within the private sector. Dörries et al. (2021) identified communication, participation and change leadership as the most critical factors for successful digital transformation. Although these two scientific approaches and their organizational context (public administration versus private sector) differ and cannot be directly compared, their empirical evidence supports and strengthens our findings.

### 5.1. Demands and Resources in Digital Transformation in the Public Administration

Among the multitude of individual and job resources identified, both employees and supervisors perceived employees’ individual technical affinity and openness towards change and digital transformation as supportive. In addition, employees perceived their own error tolerance and willingness to learn as a resource. This finding is supported by Osmundsen (Osmundsen, 2020), who stated that willingness to learn and openness to change are crucial in acquiring digital competencies and capabilities. No specific technical competences seemed to be relevant in our study, but rather soft skills and attitudes. Trenerry et al. (2021) also discussed a “growing emphasis on the importance of soft skills in technology rich work environments, i.e., communication, problem solving and creativity” (p. 7).

On the other hand, employees and supervisors perceived employees’ concerns and fears regarding digital transformation and a lack of IT skills as challenging. In addition, employees perceived the learning of new IT skills as challenging. These factors are important to consider when designing working conditions in digital transformation, as anxiety about one’s own IT capabilities can contribute to the experience of technostress, which is associated with lower well-being, productivity, and commitment to the organization (Trenerry et al., 2021). Employees’ negative attitude towards change was perceived as challenging by supervisors.

On a group level, employees and supervisors perceived social support through colleagues or supervisors as supportive. In addition, employees perceived the positive atmosphere within the team as supportive. These findings indicate that group-related resources were described as relevant in the context of uncertainty and increased demands during digital change processes. The concept of workplace social support is well studied and is related, e.g., to reduced levels of anxiety, depression, and burnout (Bowling et al., 2015; Schoofs et al., 2022); therefore, it is, seemingly, a relevant resource contributing to employees’ psychological well-being at work within digital transformation in public administration. In addition, for public administration employees clustered as digitally stressed, a lack of social support was identified as a risk factor (Wrede et al., 2021). It is noteworthy that no demands were identified that could be ascribed to group-related demands. This may reflect either an overall supportive team climate within the public administration or an active promotion of team cohesion and collaborative dynamics during digital transformation.

Considering leadership, different aspects were mentioned by employees and supervisors, reflecting both the self-perception of supervisors and employees’ leadership experience. Both groups highlighted the importance of supervisors’ open communication as supportive in digital transformation. Furthermore, employees and supervisors shared the importance of supervisors’ understanding of stressors arising in digital transformation. Supervisors experienced their own responsiveness to employees’ worries and concerns as supportive, whereas employees experienced supervisors’ understanding of different levels of IT skills as supportive, thus indicating a tension between supervisors’ intentions to provide support and employees’ expectations of concrete, capacity-sensitive leadership. In addition, employees described the importance of supervisors’ positive attitude towards change as supportive, while supervisors themselves did not emphasize the influence of their own attitudes. In the literature, different leadership skills for successful digital transformations are discussed, such as strong vision, clear goals, commitment and investment (Larjovuori et al., 2018), cooperation, team leadership, and communication skills (Philip et al., 2023).These various findings are supported by a recent systematic review that identified a current lack of a standardized conceptual framework for digital transformation leadership competencies (Raković et al., 2024). Taken together, the present findings indicate that multiple leadership skills may be relevant for supporting employees’ psychological well-being at work during digital transformation in public administration, depending on contextual organizational and cultural factors (e.g., degree of hierarchization, team size, and type of digital transformation). It should be emphasized that digital competencies of supervisors were not mentioned in the present study but might be considered a prerequisite. As supervisors skilled in digital leadership had higher levels of well-being (Zeike et al., 2019), digital leadership competencies may be relevant regarding employees’ mental-health and emotional well-being in digital transformation in public administration.

In contrast, in terms of demanding leadership behavior, employees mentioned supervisors’ negative attitudes towards change as challenging. When reflecting on their own leadership behavior, supervisors mentioned their inattentiveness towards support needs of employees as a challenge. This is in line with Deacon et al. (2023) findings, which indicate that insufficient support structures on the part of the supervisors or management were associated with employees’ rejection of newly implemented technologies.

Furthermore, supervisors expressed the moodiness of their own supervisors as a challenge. As noted by Sy et al. (2005), supervisors can transmit their emotional states to subordinates via the emotional contagion process; this may explain why this dynamic was perceived as a demand in the context of digital transformation. Overall, these findings indicate that leadership behavior can represent both a resource and a source of demand for employee well-being during digital transformation.

On an organizational level, employees perceived general counseling services and change management offers as supportive. Pacolli (2022) came to a similar conclusion, summarizing that change management measures are the basis of sustainable digital transformation; through change management offers, employees are more likely to support change.

On the other hand, both employees and supervisors experienced the insufficient planning of previous processes and the consequences (i.e., postponement of processes) as demanding. Supervisors described that decision-making bodies impeded processes, which were perceived as challenging, as well as long procedures and a lack of financial and personnel resources.

In terms of resources related to the work organization and content, employees and supervisors agreed on a multitude. Early, transparent, and participative communication before and within the process, participation of future users, individual competence development opportunities, and technical support were perceived as helpful. In terms of participation, the literature states that employees who are engaged in decision-making related to technology change react more positively to change than employees with lower levels of involvement (Schraeder et al., 2006; Sczogiel et al., 2021). In addition, lessons learned from change management in digital transformation suggest that technology acceptance can be maximized by transparent implementation, participation, and qualification of employees (Schlicher et al., 2020).

Further, the increased flexibility of work and improved efficiency of work were mentioned. These aspects can be understood as outcomes of successful digital transformation rather than working conditions. Still, these aspects are important when considering working conditions in digital transformation, as performance expectations positively influence attitudes towards digital transformation and motivation to support digital transformation (Meske & Junglas, 2021). Furthermore, the user-friendly design of applications, programs, or processes was perceived as supportive by employees. Employees and supervisors again agreed on a multitude of demands related to work organization and content; in particular, both experienced insufficient or a lack of communication and insufficient or a lack of participation as challenging. Both employees and supervisors perceived the disruption to work processes resulting from the digital transformation as a challenge. Prior research has linked perceived technological characteristics, such as usefulness or reliability, to work-related stressors like work overload (Ayyagari et al., 2011). In contrast, the present study discusses these aspects based on qualitative perceptions rather than formal measurement. Negative experiences regarding the outcome of a digital transformation are important to consider when designing future digital transformations, as negative experiences can lead to negative outcome expectation and consequently determine motivation and attitude (Meske & Junglas, 2021). In addition, supervisors experienced the integration of different user demands in the process as challenging, as well as compliance with legal regulations and data protection. In accordance with that, Mattes and Bertermann (2023) stated that legal requirements often inhibit the efficiency of digital transformation in public administration, thus underlining the demanding character of legal regulations.

### 5.2. Practical Implications

The findings of this study provide a foundation for designing demand-oriented change support and health-promoting working conditions during digital transformation in public administration. Given the broad spectrum of supportive resources and offers reported by employees and supervisors, we generally recommend implementing a comprehensive range of support measures.

The overarching practical goals should be the following:

1. Maximize identified job resources, such as transparent communication and participation, and ensure the availability of helpful support services, including competence development opportunities and technical assistance.
2. Minimize reported job demands, for example, negative attitudes of supervisors towards digital transformation.

Specifically, we propose the following:

1. Employee Involvement: Actively involve affected employees and consider their needs in participative decision-making and planning processes. Research shows that employee involvement fosters more positive reactions to technological change (Trenerry et al., 2021) and enhances openness towards change, positively impacting psychological well-being at work during transitions (Konkol et al., 2019).
2. Communication: Since many demands and resources relate to interpersonal and inter-authority communication, emphasis should be placed on improving communication within teams, between line supervisors and employees, and between higher-level and executing authorities. A centrally coordinated communication strategy is recommended, which is transparent, continuous, and participative. Importantly, communication should include bottom-up channels and feedback opportunities to empower employees.
3. Leadership: Supervisors should model a positive attitude toward digital transformation to inspire motivation and openness. They also bear responsibility for employees’ well-being during digital change by being approachable, addressing concerns, and effectively communicating team needs to higher management.
4. Support Services: Providing diverse, low-threshold, and continuous support options, such as floor walkers or multipliers, complemented by prior training sessions, can meet employees’ varying technical support needs effectively.

In summary, we advocate for implementing digital transformation processes in public administration through participative decision-making, transparent communication, and responsive leadership, complemented by a variety of supportive services. This approach aligns with Pacolli’s change management framework for sustainable and resilient digital transformation, which emphasizes employee inclusion, communication, and leadership (Pacolli, 2022). Our findings thus reinforce the critical role of structured change management in digital transformation within the public sector.

### 5.3. Research Implications

This exploratory qualitative study provides initial insights and generates hypotheses for future research on digital transformation in public administration. Given that some findings align with existing change management literature, further research is warranted to systematically investigate similarities and differences between general change management processes and digital transformation specifically.

As the present study was designed to generate initial insights and implications for the design of health-promoting working conditions in digital transformation, specifically in the context of the public administration, a more specific analysis of the generally derived factors of communication, participation, and especially leadership is indicated for further specific implications.

Further qualitative studies are encouraged to include additional perspectives, especially from employees who actively shape working conditions and influence digital transformation. Such studies should also assess the concrete needs of public administration employees in digital transformation to better tailor health-promoting interventions. Considering the relation between leadership and employee well-being, a qualitative analysis of the correspondence of line-supervisors and employees’ demands and resources could further clarify interpersonal dynamics in digital transformation as a source of resources and demands in digital transformation.

To enhance the generalizability of results, quantitative studies are necessary. In particular, quantifying the prevalence and intensity of the identified demands and resources would provide an empirical foundation for practical recommendations. Moreover, future quantitative research should examine the impact of these demands and resources on specific health outcomes, such as burnout, emotional exhaustion, somatic symptoms, and overall well-being.

### 5.4. Strength and Limitation

The strength of the present study is the generation of new in-depth insight into an emerging field of research by following an explorative, qualitative approach. By including the perspectives of employees and supervisors affiliated with a multitude of public authorities, a variety of perspectives on the research topic were provided. Most of the participants identified as female, which corresponds with the predominance of female employees in public administration. However, this sample does not represent public administration employees in terms of age, occupation, and level of education.

Several limitations should be considered when interpreting the findings. First, participants’ motivation to take part in the study is unknown; therefore, it cannot be determined whether individuals with particularly positive or negative experiences of digital transformation were more likely to participate. The absence of reported job demands related to group-level factors may reflect a genuine finding within the studied context, where digital transformation was primarily experienced at the individual and organizational level. At the same time, it cannot be ruled out that the focus of the interview questions may have encouraged participants to reflect more strongly on individual and structural aspects, which should be considered when interpreting this result. Although the sample size (*n* = 19) is relatively small, it is appropriate for qualitative research, as data collection and analysis followed an iterative process and were concluded once no new categories emerged, indicating thematic saturation. Member checking was not conducted, which may be considered a methodological limitation and represents a potential area for future research. Due to the qualitative design, the findings are not intended to be statistically generalizable. As with qualitative research more broadly, limitations include challenges in replicability and the interpretive complexity of the data (Mwita, 2022). Finally, data analysis was conducted by a single researcher; involving a second coder and calculating intercoder reliability could have further enhanced transparency and analytic rigor (O’Connor & Joffe, 2020).

## 6. Conclusions

Given the strong need for digital transformation in public administration and the simultaneous challenges related to employees’ psychological well-being at work, the aim of this study was to identify the demands and resources experienced by employees and supervisors during previous digital transformation processes, in order to derive implications for demand-oriented change support. This study contributes to a comprehensive exploration of the experienced job demands and resources, as well as personal demands and resources, in digital transformation in public administration. Overall, we conclude that implementing digital transformation within participative decisions, communication, and implementation processes, whilst supporting employees with adequate competence development opportunities and continuous IT support services, could be essential to foster health-promoting working conditions in digital transformation in the public administration.

## Figures and Tables

**Table 1:** Categories of the interview guidelines related to experiences in previous digitalization in public administration.

Employees Experienced Opportunities and Challenges	Supervisors Experienced Opportunities and Challenges
▪ Changes in work organization and content	▪ Changes in work organization and content
▪ Experienced opportunities	▪ Experienced opportunities
▪ Experienced challenges	▪ Experienced challenges
▪ Support factors	▪ Support factors
▪ Perception of leadership	▪ Reflection of own leadership behavior
▪ Perception of organization	▪ Perception of organization

**Table 2:** Participant characteristics of employees and supervisors in public administration (*N* = 19).

Participant Characteristics *N* (%)			
Gender	Total (*N* = 19)	Supervisors (*n* = 11)	Employees (*n* = 8)
Male	6 (31.6)	6 (54.5)	1 (12.5)
Female	12 (63.1)	5 (45.5)	6 (75)
Non-binary	1 (5.3)	0	1 (12.5)
Age (in years)			
21–30	3 (15.8)	1 (9.1)	2 (25)
31–40	2 (10.5)	1 (9.1)	1 (12.5)
41–50	6 (31.6)	5 (45.5)	1 (12.5)
51–60	7 (36.8)	3 (27.3)	4 (50)
61–70	1 (5.3)	1(9.1)	0
	*M* = 46.70 (*SD* = 11.24), range: 26–65 years		
Employment			
Full-time	16 (84.2)	9 (81.2)	7 (87.5)
Part-time	3 (15.8)	2 (18.2)	1 (12.5)
Weekly working hours (in hours)			
<20	1 (5.3)	0	1 (12.5)
≥20–35	2 (10.5)	2 (18.2)	0
>35	16 (84.2)	9 (81.2)	7 (87.5)
Employment contract			
Unlimited employment	9 (47.4)	2 (18.2)	7 (87.5)
Civil servants	10 (52.6)	9 (81.2)	1 (12.5)
Highest education ^1^			
Vocational training	5 (29.4)	2 (22.22)	3 (37.5)
Bachelor’s degree	0	0	0
Master’s degree or higher	12 (70.6)	7 (77.78)	5 (62.5)
Working experience in public administration (in years)			
<1	0	0	0
≥1–3	5 (26.3)	3 (27.3)	2 (25)
≥3–10	8 (42.1)	3 (27.3)	5 (62.5)
>10	6 (31.6)	5 (45.4)	1 (12.5)
	*M* = 9.26 years (*SD* = 10.22), range: 1.5–40 years		
Number of team members led ^2^			
1–5	-	5 (45.4)	0
6–10	-	2 (18.2)	0
11–15	-	1 (9.1)	0
15–30	-	2 (18.2)	0
>30	-	1 (9.1)	0
	*M* = 14 people (*SD* = 20), range: 1–70		

^1^ *n* = 17, as there is missing information about the educational background of two supervisors. ^2^ Is referring to supervisors only.

**Table 3:** Overview of identified job demands and job resources. Key themes are highlighted in bold; additional illustrative quotations are provided in the Supplementary Material (Table S2).

	Resources in Digital Transformation	Demands in Digital Transformation
**Personal factors**	▪ Technical affinity ^e, s^ ▪ **Openness towards change and digital transformation ^e, s^** ▪ Error tolerance and willingness to learn ^e^	▪ Personal concerns and fears ^e, s^ ▪ **Negative attitude towards change ^s^** ▪ Learning new IT Skills ^e^ ▪ Lack of IT skills ^e, s^
**Group factors**	▪ Positive team atmosphere ^e^ ▪ Social support ^e, s^	▪ no group related demands were mentioned
**Leadership behavior**	▪ **Positive attitude towards change ^e^** ▪ Open communication ^e, s^ ▪ Understanding of different IT skills ^e^ ▪ Awareness of stressors for employees in digital transformation ^e, s^ ▪ Responsive to worries, concerns, questions ^s^ ▪ Empathy, Patience ^s^	▪ **Negative attitude towards change ^e^** ▪ Inattentiveness to support needs of employees ^s^ ▪ Moodiness ^s^
**Organizational factors**	▪ General counseling service ^e^ ▪ General responsible contact person ^s^	▪ Decision-making bodies impede processes ^s^ ▪ Insufficient planning ^e, s^ ▪ Long procedures ^s^ ▪ Lack of resources ^s^
**Work organization and content**	▪ **Participation ^e, s^** ▪ **Early and transparent communication ^e, s^** ▪ **Improved efficiency of work processes ^e, s^** ▪ Increased flexibility of work ^e, s^ ▪ Use friendly design ^e^ ▪ Individual competence development ^e, s^ ▪ Technical support ^e, s^	▪ **Insufficient or lack of participation ^e, s^** ▪ **Insufficient or lack of communication ^e, s^** ▪ **Digital transformation impedes work processes ^e, s^** ▪ Integration of different demands ^s^ ▪ Legal regulations and data protection ^s^

^e^ = indicates that employees mentioned the respective demand or resource; ^s^ = indicates that supervisors mentioned the respective demand or resource.

## Data Availability

The data generated and analyzed during this study are not publicly available to protect the anonymity of participants. Upon reasonable request, the data are available from the corresponding author.

## Note

1 In the hierarchical context of public administration, “superiors” refers to the direct hierarchical supervisors of the participating supervisor, representing a higher organizational level.

## Supplementary Materials

The following supporting information can be downloaded at: https://www.mdpi.com/article/10.3390/bs16020187/s1, Table S1: Consolidated criteria for reporting qualitative studies (COREQ): 32-item checklist; Table S2: A selection of further relevant interview quotes.

## Abbreviations

The following abbreviations are used in this manuscript:

COREQ	Consolidated criteria for reporting qualitative research
ICT	Information and communication technologies
EU	European Union
GDA	Joint German Occupational Safety and Health Strategy
JD-R	Job demands-resources
PCI	Problem-centered interview

## Appendix A

**Table A1:** Structure of the interview guidelines used in a problem-centered interview.

Interview Phase	Communication Strategies	Contents
1. Introductory phase	N/A	▪ Information on the study ▪ Standardized short questionnaire on sociodemographic, work experience, and work-related information (paper-and-pencil based)
2. Job demands and resources in previous digital transformations	Strategies to generate storytelling	Introductory question: ▪ “Have you ever experienced a digitization process at your current position or a previous employment within the public administration? How did you experience the change? General exploration, open questions: ▪ Open questions about experienced demands and resources (e.g., aspects in previous digital transformations that were supportive and aspects that had adverse effects on the process) Ad hoc questions: ▪ Questions on specific areas, e.g., social support, communication, involvement, leadership, organizational behavior
	Strategies to generate comprehension	▪ Reflection of the participants’ previous statements if unclear
3. Final phase and farewell		Final question: “Is there anything I haven’t asked you or that we haven’t discussed that is relevant to this topic from your perspective?” ▪ Clarify any open questions of participants ▪ Appreciation of participation
