# Supplementary material for: Job Demands and Resources During Digital Transformation in Public Administration: A Qualitative Study
Source: Behav Sci (Basel). 2026 Jan 27;16(2):187. doi: 10.3390/bs16020187 (PMC12938550; doi:10.3390/bs16020187)
Supplement: Supplementary file 1 [file behavsci-16-00187-s001.zip › Supplement Material S2.pdf]

Table S2. A selection of further relevant interview quotes (without claiming to be exhaustive)

**1. Resources perceived by employees and leaders in previous public administration digital transformation**
**Personal resources**
Technical affinity
"You definitely need a digital competence, (...), so sometimes it's also about this confidence, 'Okay, I'll try it out now', where I've experienced other colleagues who were a bit hesitant, 'Oh gosh, if I press it now'. I know that, 'Okay, you can try it, you can undo it, no problem'. That's exactly why I think I got through the time quite well." (Participant 17, female leader, age 21-30)
**Group resources**
Social support
"And in general, I have to say that the collegial exchange, i.e. the team spirit, was really great at the time. That's how I perceived it, that people supported each other. I was perhaps able to provide technical help where others were able to provide legal assistance in some way, because you really have to think about it, if you're not sure, 'is the system doing it right'; then, you have to know the legal basis really well. And you also have to be able to do the math, so that you can work out, 'is that right', by hand if necessary. And of course, the colleagues who have been working at [name of authority] for, I don't know, 20 years were great. So, they always supported each other according to their skills." (Participant 17, female leader, age 31-40)
**Leadership behavior**
Awareness of stressors for employees in digital transformation
"And then also to make it clear that they, and these were actually all female colleagues, would then be relieved of their normal daily duties, so that appropriate care would be taken." (Participant 12, female leader, age 61-70)
**Resources related to work organization and content**
Improved efficiency of work
"You don't need all that paper anymore, you don't have to wait, and you know who to talk to if something gets stuck somewhere. You can see who the last person was to work on it and where it is. You can read the notes that people write. In the past, something was written by hand and then you sat there wondering, 'What did the department head write about this? I can't read it' (...). It's transparent, it's traceable."(Participant 13, female employee, age 51-60)
Flexibility of work
"You can work much more flexibly. So, it is possible. We were already able to work well from home during the pandemic.You always had to prepare a little and gather a few things that I could do from home, because you need a lot of the [work content] and have to look into it. And now, I think for me as a [field of activity], because I have to look at the [work content] even less, because I have fewer personnel cases and the others then approach me and they actually have the most important key points. So, theoretically, I could stay in my home office for a week. So, this mobile working and flexible working has already changed significantly as a result, and I would also say improved." (Participant 19, female employee, age 21-30)
"You can work much more flexibly. We were also able to work from home during corona (COVID-19 pandemic). You always had to prepare a bit and gather a bit of information about what I could do when working from home, because you need to look at the files a lot. And now it's. I think for me as a [field of activity], because I have to look at the files even less because I have fewer personnel cases and the others contact me and they actually have the most important key points. In theory, I could also stay in my home office for a week. So, this remote working and flexible working has changed significantly and I would also say improved." (Participant 19, female employee, age 21-30)
Participation
"We can contribute our comments there and they are also tried to implement these. So, from my point of view, it worked very well." (Participant 14, female employee, age 51-60)
Technical support
"I also noticed at the beginning that the program kept freezing up and I couldn't use anything at all and then I asked IT myself what the problem might be. It's just an old problem and I had to live with technical bugs (…). So, if I had any major technical problems that suddenly occurred, IT was able to help me in some places and so everything was fine so far." (Participant 4, female employee, age 21-30)

Table S2. A selection of further relevant interview quotes (without claiming to be exhaustive)

Individual competence development
"I wasn't involved in the development of the program, but before it went online, so to speak, we received a lot of training, including live training, so that we could do everything the way it really is. From my point of view, it worked very well." (Participant 14, female employee, age 51-60)
And that's why I think it was good that we had the opportunity to take part in a central information and training event before the launch. That was our wish. The [name of authority] complied with this request and offered us a training date with a representative from this company, so to speak. That was very open. She didn't just reel off her program but presented the solution. Not in the real application, but in a training environment, so to speak, but that was fine to develop an understanding of the system and the application. So, we didn't feel that we were being left alone with a new solution that we had to use, but that we were being prepared for it at our request. This was not actually planned as part of the implementation strategy. So, you can criticize that or not. But when the request was put forward, it wasn't rejected, it was okay by those who thought they needed training. Then we'll do it. And we didn't need four hours of the entire morning that was scheduled for it, but after an hour and a half or two hours, everyone had understood it and felt sufficiently and thoroughly informed to try it out and apply it themselves in real operations from September or October, so to speak. It works quite well." (Participant 26, male leader, age 51-60)
**2. Demands perceived by employees and leaders in previous public administration digital transformation**
**Demand related to work organization and content**
Insufficient planning
"Exactly the same with the [program], which should have been there two years ago. It has always been postponed. Now it's been moved to unknown because the entire department responsible for the [program] in [name of authority], or almost the entire department, has disbanded. This has caused everything to shift. Some colleagues are already jokingly saying that they won't live to see it until they retire and one colleague still has five years left. So, it could be better." (Participant 16, male employee, age 41-50)
"And the other thing is, how the process is set up, how well are the employees involved (…) is the process centrally managed by [name of authority]. And I would say there was room for improvement, I really have to say that. There was some form of change management team but honestly, we were pretty much on our own and felt we could never overcome the problems. And there were a lot of problems. I think they also misjudged this at the beginning, they really overdid it for my taste and that made the process so much more difficult. At the beginning there was a good willingness among everyone and after a while it just meant that nobody wanted to continue with this process." (Participant 15, male leader, 61-70)
Digitalization impedes work
"I think we've had a new form since [date], which you always had to download from the [name portal]. And it always didn't open properly. I kept getting calls from employees saying that they couldn't open it because it wouldn't open in [name of program], but it would open in [name of program] and not in the new Internet, but in the old one. And a lot of people couldn't cope with that and I then turned to [name of authority] to see if there was any way to change it. But that couldn't be done. So, it went on for a year or so that it didn't work properly. And if you provide a form like that and it doesn't work, then it's pretty tedious. I've probably been asking and trying and trying again and again, I don't know how long, to find out why it doesn't work and whether it does work and whether I can find another solution, and I found that quite tedious. And something like that shouldn't really happen." (Participant 13, female employee, age 51-60)
Insufficient communication
"Because the interfaces are not clear and the right hand doesn't know what the left hand is doing; even here in our own [authority] things are running parallel and nobody knows about it." (Participant 02, female leader, age 41-50)
"Although an email was sent to all employees across the city almost two years ago, I still didn't reach all employees. I still get requests these days, 'Yes, I need another statement', or 'I need my payroll tax evaluation from last year', where I say,'[name program] all data is in there'." (Participant 16, male employee, age 41-50)
Insufficient participation
"But as I said, many of them simply felt left alone and were a little outraged and frustrated. It was a mix of feelings, but they didn't like it. Oh well, I think they didn't really give the software a chance. In itself, it was mostly very user-friendly, which is also important. I actually find it very intuitive, but because they were left alone so much from the start, they didn't really give the whole thing

Table S2. A selection of further relevant interview quotes (without claiming to be exhaustive)

a chance." (Participant 4, female employee, age 31-40)
Integration of demands
"I find it very exciting that, since this pilot phase, I have basically been working through sticking points that were discussed in advance before the introduction, because there is always this issue: the authorities have to agree on a common denominator, and no matter how similar the legal bases are, the processes in the individual institutions are still very different. That was an issue in the run-up to the launch, and I think it continues to be an issue today because we are still talking about some things that were discussed in advance but on which we were unable to agree at the time." (Participant 10, female leader, age 41-50)
